# The Therapeutic Alliance between Pomegranate and Health Emphasizing on Anticancer Properties

**DOI:** 10.3390/antiox12010187

**Published:** 2023-01-12

**Authors:** Panagiota D. Pantiora, Alexandros I. Balaouras, Ioanna K. Mina, Christoforos I. Freris, Athanasios C. Pappas, Georgios P. Danezis, Evangelos Zoidis, Constantinos A. Georgiou

**Affiliations:** 1 Department of Biotechnology, Agricultural University of Athens, 11855 Athens, Greece; 2 Department of Chemistry, National and Kapodistrian University of Athens, 15784 Athens, Greece; 3 Laboratory of Nutritional Physiology and Feeding, Department of Animal Science, Agricultural University of Athens, 11855 Athens, Greece; 4 Chemistry Laboratory, Department of Food Science and Human Nutrition, Agricultural University of Athens, 11855 Athens, Greece

**Keywords:** animal, antioxidant, cancer, flavonoids, health, polyphenolic compounds, pomegranate

## Abstract

Pomegranate is a fruit bearing-plant that is well known for its medicinal properties. Pomegranate is a good source of phenolic acids, tannins, and flavonoids. Pomegranate juice and by-products have attracted the scientific interest due to their potential health benefits. Currently, the medical community has showed great interest in exploiting pomegranate potential as a protective agent against several human diseases including cancer. This is demonstrated by the fact that there are more than 800 reports in the literature reporting pomegranate’s anticancer properties. This review is an update on the research outcomes of pomegranate’s potential against different types of human diseases, emphasizing on cancer. In addition, perspectives of potential applications of pomegranate, as a natural additive aiming to improve the quality of animal products, are discussed.

## 1. Introduction

Pomegranate (*Punica granatum *L.) is a tree belonging to the *Punicaceae* family. It is native to central Asia and its origin is considered to be in Iran. However, thanks to its high adaptability to a variety of soil and climate conditions, it is nowadays cultivated in many different geographical regions all over the world [1,2,3,4,5]. There are two major pomegranate tree types: edible and ornamental ones. The edible tree type includes several cultivars with sour, sour-sweet, and sweet fruits according to juice taste or organoleptic properties. On the other hand, cultivars of the ornamental type produce large attractive flowers [6]. Throughout the years, many cultures have used pomegranate tree parts, including but not limited to pomegranate leaf (PL) or leaf extract (PLE) and the flowers (PF) as a remedy in traditional medicine [7]. Research on the antioxidant, phytochemical and bioactive compounds present in pomegranate fruit generated considerable interest on several potential uses of the fruit beyond its primary use of fresh consumption [1,6,7]. Studies on pomegranate fruits usually focus on the pomegranate peel (PP) also known as pericarp or its extract (PPE), pomegranate seed (PS), pomegranate juice (PJ), extract from peel and seeds or the whole fruit (PE) and pomegranate seed oil (PSO) [7,8]. Pomegranate by-products such as seeds and peel comprise of 12% and 50% of the whole fruit, respectively [9]. Given that pomegranate global production is about 4 million tons and at the same time pomegranate juice processing generates large amounts of wastes composed mainly of peel (~78%) [10], there is a huge potential both for material valorization and for reclaiming wasted bioactive compounds.

Its popularity has risen during the last few years, mainly due to its high content in antioxidant phytochemicals and bioactive compounds. Pomegranate compounds have attracted the interest of many scientists mainly due to their antimicrobial and antioxidant activity [11,12]. Pomegranate is an important source of several bioactive compounds including but not limited to dietary fiber, vitamins, minerals, unsaturated fatty acids, phenolic compounds, alkaloids, triterpenes, and sterols. Similarly, the pomegranate seed oil contains palmitic, stearic, oleic, linoleic and four isomers of linolenic acid with the most important isomer of linolenic acid to be punicic acid [8]. These bioactive compounds have been suggested to exert numerous beneficial health activities making pomegranate a possible functional food [13]. Thus, they are responsible for the wide range of its nutritional, medicinal, antioxidant, and antimicrobial benefits [14,15,16]. However, it should be mentioned that variations of phenolic contents and antioxidant activity may exist, and these variations may be attributed to the different growth conditions of pomegranate tree and/or to different parts of the fruit [9].

## 2. Pomegranate and Obesity, Insulin Resistance, and Cardiovascular Diseases

Nowadays, in global literature, there is a variety of reports concerning the healing properties of pomegranate for an abundance of diseases and disorders, including obesity, diabetes, aging, and inflammation. Specifically, it has been proven that the natural compounds contained in the pomegranate can be used for the treatment of obesity [17]. Pomegranate seed-oil, which is rich in punicic acid, combined with exercise provided a greater effect on immune function in high fat diet treated rats compared to either exercise or pomegranate supplementation alone, indicating potential inhibition of inflammation and decrease of oxidative stress [18]. In addition, the consumption of 800 mg/kg PLE by obese mice reduced the gain of weight and the energy intake [19]. More specifically, a reduction in energy intake, glucose levels, and mice body weight was noted after 5 weeks of PLE treatment, showing that PLE may inhibit the development of obesity and hyperlipidemia in high-fat diet induced obese mice. Considering that diabetes is related to increased oxidative stress and atherosclerosis development, PJ consumption by diabetic persons did not influence diabetic parameters per se, but rather resulted in antioxidant effects on blood parameters, which could contribute to suppression of atherosclerosis development in these patients [20]. Under this context, the antidiabetic effects of PP active fraction were reported to be related to α-glucosidase inhibition and enhanced glucose uptake [21]. In diabetic mice, it has also been found that the intake of ellagic acid protected against glycative and inflammatory progression. These results suggest that ellagic acid supplementation might be helpful for the prevention or treatment of diabetes-associated kidney diseases [22]. Similarly, in mice, type 2 diabetes risk was reduced following consumption of PSO indicating an improvement in insulin sensitivity and beneficial effects on weight, leptin, and adiponectin concentrations [23]. However, a systematic review and meta-analysis on the effects of pomegranate supplementation on metabolic status in patients with type 2 diabetes mellitus (T2DM), revealed that there were no significant favorable effects of pomegranate supplementation on metabolic parameters in patients with T2DM indicating that more well-designed, large scale randomized controlled clinical trials with longer duration are needed [24].

Pomegranate exhibits anti-aging properties, due to the presence of the antioxidant phenolic acids, flavonoids, and other polyphenolic compounds. Additionally, it has anti-inflammatory properties, since it has been proved that punicalagin, punicalin, strictinin A and granatin B (hydrolysable tannins) significantly reduce the production of nitric oxide and PGE2 (prostaglandin E2), inhibiting in this way the expression of various pro-inflammatory proteins [25]. Several clinical studies have shown that pomegranate may exert beneficial effects on the cardiovascular system including improved endothelial function and lower blood pressure [26,27,28]. Pomegranate juice can enhance blood flow to the heart and its methanol and ethanol extracts showed a strong anti-inflammatory activity, with IC50 6.20 ± 0.17 and 6.83 ± 0.37 mg/L, respectively [29]. However, a systematic review and meta-analysis on the effects of pomegranate juice on vascular adhesion factors revealed no significant effect on intercellular adhesion molecule 1, vascular cell adhesion molecule 1, and E-selectin. However, it indicated that pomegranate juice can significantly reduce interleukin-6 [30]. Thus, in future, prospective controlled randomized trials with longer intervention duration may help to further elucidate pomegranate juice effects. Under this context, another systematic review and meta-analysis on the effects of pomegranate juice on selected biomarkers of inflammation and vascular dysfunction revealed that in adults several factors can be affected by pomegranate namely high-sensitivity C-reactive protein, serum interleukin-6 and the pro-inflammatory cytokine tumor necrosis factor alpha [31]. However, the same meta-analysis indicated that the effects of pomegranate supplementation on C-reactive protein, E-selectin, intercellular adhesion molecule, vascular cell adhesion protein and malondialdehyde (MDA) were not significant [31].

## 3. Pomegranate and Cancer

Recently, the medical community has showed increasing interest in using pomegranate to treat cancer. This is clearly illustrated by the fact that there are more than 800 reports in the literature concerning its anticancer properties. Herein, we try to summarize the results for the most frequent types of cancer namely breast, prostate, skin, lung, ovarian, oral, pancreatic, and liver. 

### 3.1. Breast Cancer

Breast cancer is the most common type of cancer among women. The American Cancer Society reported that in 2019, 2670 men and 268,600 women were diagnosed with invasive breast cancer [32] while in 2022, 287,850 new cases of invasive breast cancer are expected to be diagnosed in women in the U.S. [33]. The results of in vitro and in vivo experiments demonstrate the healing and protective effect of pomegranate against breast cancer.

Banerjee et al. found that (PE) enhanced the inhibitory effect of Tamoxifen, a selective estrogen receptor modulator used to prevent breast cancer in women, on resistant breast tumors [34]. On a mouse mammary cancer cell line, designated as WA4, administration of PE containing 37.5% ellagitannins and 2.7% ellagic acid inhibited their proliferation [35]. In addition, incubation of BT474 and MDA-MB-231 epithelial, human breast cancer cells with polyphenols or PE resulted in a reduction in their survival, without affecting the normal, non-tumorigenic MCF-10F and MCF-12F epithelial cells [36,37,38]. In BT474 cells the tumor size decreased, while the growth of MDA-MB-231 and SUM149 cells was reduced by 67% and 24%, respectively [36,37]. Shirode et al. proved that 20 μg/mL of PE after 72 h and 96 h incubation with MCF-7, decreased their growth by 30% and 35%, respectively, induced apoptosis and arrested cell cycle between G2/M phases [39]. MCF-7 breast carcinoma fibroblasts were shown to be the most sensitive cells to (PP) applied as a potential anticancer agent [40]. Furthermore, in MCF-7 cells, apoptosis and reduction in proliferation have been observed, after the addition of 200–300 μg/mL of PE for 48–72 h [41], while in a different study the acetonic pomegranate peel extract, after 48 h of incubation, exerted high antiproliferative efficiency against MCF-7 cells, as indicated by the IC50 value (8.15 μg/mL) [42]. When pomegranate extract, rich in ellagic acid and punicalagin, was added to the neoplastic mammary epithelial HMLER and breast cancer Hs578T cells, a reduction in their ability to regenerate was observed [43]. Extracts rich in punicalagin and cyanidin chloride exhibited a strong cytotoxic activity on MCF-7 cells, after 24 h incubation, with an IC50 value of 49.08 μg/mL [44]. Additionally, the anticancer activity of black PE, a rare pomegranate cultivar, was evaluated. The results showed that the extract induced the cell death pathway in more than 70% of the cancer cell lines MCF-7 and BT-20 [45]. In a study conducted by Bagheri et al., it was shown that (PPE) suppressed the migration and invasion of MDA-MB-231 cells at concentrations of 25, 50, 100, 250, 500 and 1000 μg/mL, while at higher concentrations induced apoptosis [46]. Peel extract (5 μg/mL) was found to inhibit proliferation of MCF-7 fibroblast cells by 83.7%, in accordance with other results showing that polyphenols originating from PSO or PJ hinder cancer cells division [47,48]. In addition, homogenized pomegranate peel (500 μg/mL) decreased MCF-7 proliferation by 12–93% and MDA-MB-453 by 83% [49]. Methanolic PE moderated the growth of MCF-7 and inhibited their proliferation, which was stimulated by 27-hydroxycholesterol and associated with the development and metastasis of breast cancer tumors [50].

In addition to the most tested pomegranate extracts, other constituents of the pomegranate or its active compounds have been studied for possible anticarcinogenic properties. Pomegranate juice (PJ) at a concentration of 1 mg/100 mL inhibited 56% of estrogens action [51]. In addition, Rocha et al. reported that the addition of juice or luteolin with punicic acid and ellagic acid to MDA-MB-231 and MCF-7 cells inhibited their growth and stimulated apoptosis, without affecting healthy cells [52]. These compounds also reduced the chemotaxis of cells through SDF1α (stromal cell-derived factor 1, a chemokine), implying that they affect metastasis [52]. Fermented juice polyphenols (100–1000 μg/mL) inhibited the action of aromatase by 60–80%, by 79% the 17-β-hydroxysteroid dehydrogenase type 1 and by 38% the action of 7.12-dimethylbenz (α) anthracene, which causes damage to precancerous stem cells [53]. MCF-7 cells also present a significant sensitivity to the presence of γ-linoleic acid [54]. In another experiment, Adams et al. studied six ellagitannin derivatives and found that all of them inhibited the action of aromatase on MCF-7aro cells [55]. In addition, delphinidin at a concentration of 50 μg/mL reduced by 50% the proliferation of six different cell lines [56]. The hydrophilic fragments of pomegranate oil suppressed the viability of MDA-MB-231 and MCF-7 cells, after 24 h incubation, without causing much apoptosis [57]. It is interesting to note that non-edible parts, such as roots, tree bark and fruit, also inhibited the proliferation of MCF-7 by 94% [58]. 

In recent years, attempts have been made to encapsulate the bioactive compounds of pomegranate in nanoparticles, in order to optimize their bioavailability and targeted transport. Extracted nanoparticles exhibited cytotoxic activity in MCF-7 cells and induced apoptosis in a dose-dependent manner [59]. Biodegradable Pt nanoparticles (PtNPs), in which pomegranate extract was encapsulated, reduced the proliferation of MCF-7 by 80% after 72 h of incubation and the IC50 value was 17.84 μg/mL after 48 h of incubation [60]. In addition, when pomegranate extract was encapsulated in Ag nanoparticles (AgNPs), it decreased the proliferation of MCF-7, with an IC50 value of 12.85 μg/mL [59,61] According to the results of two independent experimental studies, the addition of AgNPs loaded with PE (100 μg/mL) to MCF-7 cell cultures exhibited the potential to inhibit their survival by 50% [62,63]. Moreover, PE encapsulation in AgNPs resulted in inhibition of MDA-MB-231 metastatic breast cells viability, recording an IC50 value of 72.314 μg/mL [64]. Lactate dehydrogenase (LDH) and 3-(4,5-dimethylthiazol-2-yl)-2,5-diphenyltetrazolium bromide (MTT) cytotoxicity assays conducted by Taherian et al. confirmed that chitosan-coated magnetic nanoparticles loaded with PE could significantly eradicate 4T1, MBA-MB-231 and NIH/3T3 breast cancer cells, without affecting normal cell lines [65]. Finally, the encapsulation of peel and folic acid in Au nanoparticles (AuNPs) showed cytotoxic action against MCF-7 cells. All the above indicate the possibility of alternative drug transport for therapeutic purposes [66]. 

### 3.2. Prostate Cancer

Prostate cancer is the second most common type of cancer among men and the fourth most common type overall. According to in vitro studies, PJ affected the viability of DU145 prostate cells in a dose- and time dependent manner, without affecting normal cells [67]. The addition of juice also prevented the growth of PC-3, DU145, and LNCaP cells, induced apoptosis and prevented their metastasis and chemotaxis, stimulated by SDF1α [68]. The presence of the bioactive compounds luteolin, ellagic acid, and punicic acid in the juice, not only acted against migration and growth of PC-3, DU145, and LNCaP cells, but also led the cells to a strong adhesion to the substrate, increased the levels of miRNAs that suppress tumors, and reduced those that are related to oncogenesis [69]. The ellagic acid of the juice led to a decrease in LNCaP cells, reduced their growth by 35% and arrested the cell cycle [70]. The peel extract reduced the viability of the cells by less than 40% and the maximum reduction in mean tumor size was 79.3% [71]. A concentration of 50 μM ellagic acid mitigated PC-3 proliferation and changed their morphology after 72 h incubation [72,73]. Ellagic acid and punicalagins, at a concentration of 3.4% and 37–40%, respectively, reduced the growth of LNCaP, LNCaP-AR, and DU145 cells [74]. In studies conducted by Adhami et al. using the oil, fermented juice, and phenols from the pomegranate’s pericarp, was observed that all ingredients suppressed the proliferation of LNCaP, PC-3, and DU145 cells, while they did not affect normal cells [75]. The pericarp extract showed anti-proliferative and proactive action, inhibited tumor growth, and reduced prostatic specific antigen (PSA) [75]. Incubation of extract with LaPC4 cells, suppressed their proliferation by 20%, while the combination of extract (from skin and arils minus seeds) and IGFBP-3 decreased it by 30% indicating a synergistic action, which also caused apoptosis [76]. It has also been found that extract containing 37% ellagitannins and 3.5% ellagic acid suppressed the proliferation of LNCaP cells, as well as HUVEC endothelial cells, under conditions with or without O_2_ [77]. Whole fruit polyphenols, the pericarp, and the seed oil presented anti-proliferative properties against LNCaP, PC-3 and DU145 cells [78]. Their presence prevented PC-3 cells from invading through Matrigel, while the seed oil significantly increased the G2/M ratio in DU145 cells [77]. Cytotoxic studies have shown that the fruit seed extract had a strong effect on PC-3 cells as it suspended them, by 85.37% [79]. In addition, the incubation of PC-3 with 250 and 500 μg/mL of pomegranate extract reduced their viability by 84.16% and 84.00% respectively [47,80]. Other components of pomegranate that have a cytotoxic effect are caffeic acid, punicic acid, punicalagin and luteolin. In particular, ellagic acid, caffeic acid, punicic acid, and luteolin when added together, equally on PC-3 cultures, suspended their invasion through Matrigel, indicating their synergistic action [81]. Punicalagin in concentrations of 10, 50 and 100μM reduced the proliferation of LNCaP cells by 30%, 60% and 70% and of PC-3 cells by 28%, 52% and 55%, respectively, after 96 h incubation and caused apoptosis [82]. Finally, urolithin A significantly affected DU145 and PC-3 cells, with the former being more sensitive [83].

In addition, there are many in vivo studies conducted, to study the cytotoxic properties on prostate cells. Mice with severe combined immunodeficiency (SCID) infected with LAPC4 cells were fed with PPE, containing 37–40% punicalagins and 3.4% ellagic acid [84]. The results showed that tumor growth, after mouse castration, stopped, while NF-κB activity decreased [84]. In addition, castrated mice fed with pomegranate for 1 week did not develop cancer in LAPC4 cells, while all tumors had a smaller size than controls [84]. In an experiment, consumption of an extract rich in ellagic acid and urolithin A, from mice with prostate cancer, reduced the growth of LAPC4, LNCaP, DU145, and 22RV1 cells [85]. In addition, pomegranate peel extract caused a reduction in the proliferation of PC-3, DU145 and TRAMP-C1 mouse cell lines, leading to apoptosis [86]. In addition, 64 g luteolin, ellagic acid, and punicic acid were administered to SCID mice with prostate cancer in a 1:1:1 ratio and a reduction in tumor volume and prevention of metastasis were observed [87].

There are several publications related to the application of pomegranate in clinical trials. In phase II clinical trials, it has been shown that treating patients with pomegranate extract slowed PSA doubling time without any side effects [88,89]. In another clinical study, consumption of 8 oz (236.5 mL) of juice by men with prostate cancer significantly increased PSA doubling time, from 15 to 37 months, without any side effects or metastasis [90,91,92,93,94]. Moreover, daily consumption of pomegranate extract (1000 mg), for 12 months, by patients decreased androgen rate signaling in prostate tumors, as well as the expression of biomarkers related to oxidative stress [89]. As reported by a systematic review, nutritional interventions with pomegranate or other bioactive substances present in green tea, broccoli, and turmeric all demonstrated beneficial effects [95]. Finally, men with increasing PSA who consumed juice after radiation or surgery showed an increase in the mean PSA doubling time from 15 to 54 months, an increase in cell apoptosis and a decrease in cell proliferation [96].

### 3.3. Skin Cancer

Skin cancer is the most common type of cancer among men and women, while its worldwide incidence is 40%. Exposure to UV-B and UV-A radiations is the main cause of skin cancer, as these types of radiation activate the action of various kinases, the NF-κB factor, as well as other complexes involved in carcinogenesis. However, in vitro and in vivo studies have shown that the bioactive constituents of pomegranate have a chemoprotective effect.

Studies conducted on the normal human epidermal keratinocytes (NHEK) cell line showed that the addition of PE prevented the phosphorylation of the ERK1/2 kinases, STAT-3, and kinase AKT/Mtor/p705, which are activated after exposure to UV-A [97]. In the same studies, it was observed that PE arrested the cell cycle in G1 phase and prevented the nuclear shift. The extract had a protective effect on HaCaT cells, against photoaging and oxidative stress, which are caused by UV-B [98,99]. In NHEK cells, PE prevented the phosphorylation of p38 kinases, the JNK1/2, which is stimulated by UV-B radiation, the c-Jun complex, which triggers the action of other kinases and the activation of the NF-κB factor [25,100]. The results were depended on the concentration and application time of the extract. It has also been found that PE inhibits the synthesis of melanin, as well as the proliferation of melanocytes due to the inhibition of the tyrosinase action [101]. In B16710 cell lines of melanoma, the extract from the peel of black pomegranate, which contains the highest percentage of flavonoids, was toxic, with little effect on healthy HUVEC cells [102]. At the same time, according to studies, the phosphorylation of MAPKs and MMPs-1,2,7,9 in HaCaT cells was significantly reduced, while it enhanced the viability of normal cells [98]. Specifically, the action of UV-B was inhibited when 20 mg/L of extract were added [103]. Finally, PSO has also been found to help in cancer prevention caused by UV-B radiation [104].

Furthermore, in vivo studies have shown the effectiveness of pomegranate on skin cancer. SKH-1 hairless mice, diagnosed with skin cancer, consumed PE with their water and showed a significant decrease in tumors, CPDs, cancer cells and 8-oxodG (8-dihydro-2′-deoxyguanosine an index of nuclear DNA damage) [105,106,107]. Mice, in which a tumor was formed by induction with 7,12-dimethylbenz(a)anthracene, (DMBA) an immunosuppressant and powerful laboratory carcinogen, the application of oil from pomegranate fruit reduced the rate of tumor formation and their multiplicity, as the average number of tumors in mice used as witnesses was 20.8, while in treated mice 16.3 [108]. In these mice, the addition of pomegranate fruit extract (PFE) to the skin, before adding 12-O-tetradecanoylphorbol-13-acetate (TPA, a potent tumor promoter) reduced the appearance of edema and inhibited the action of ornithine decarboxylase (ODC) enzyme, which was induced by TPA. In addition, it promoted the formation of skin volume [109], the expression of COX-2 and the phosphorylation of ERK1/2 and NF-κB/p65. In addition, application of transdermal emulsion containing pomegranate extract loaded solid lipid nanoparticles (PE-SLNs) to the skin of mice with Ehrlich ascites carcinoma (EAC) significantly reduced tumor size compared to controls [110]. Finally, in mice with skin cancer, the synergistic effect of the fruit extract with diallylsulfide (an organosulfur compound derived from *Allium* plants) was confirmed [111]. 

### 3.4. Lung Cancer

Most studies related to the inhibitory effect of pomegranate on lung cancer have been performed on A549 cell lines [112]. Specifically, according to literature, when extracts from the whole fruit were added to the A549 cancer cells, they reduced their viability, and at the same time no toxic effects were observed on normal, bronchial cells [113,114]. In these cells, cell cycle was arrested in the G0 to G1 phases, due to reduction in the expression of p21 and p27 kinases, as well as cyclins D1, D2, E, and cdks-2, -4, and -6 [113,114]. It was also found that the extract inhibited various biochemical pathways, such as NF-κB, PI3K/AKT and MAPK [112,115,116], as well as c-met and angiogenesis [114]. In addition, experiments have shown that the addition of 250 μg/mL of PPE to A549 cells suppressed their growth by 80% [79]. When pomegranate fruit extracts in concentrations ranging from 50–150 μg/mL were added to A549 cells, their survival rates were reduced by 47%, without affecting normal NHBE bronchial cells [117]. In addition, these extracts affected WAF1/p21 and KIP1/p27 cyclin-dependent kinase inhibitors, which are involved in the transition from the G1 phase to S, and reduced the expression of cyclin D1, D2, E, cdk2, cdk4, and cdk6 in a dose-dependent manner [116]. Ellagitannins contained in pomegranate suppressed the anti-apoptotic factor Bcl2 in a dose-dependent manner and led to apoptosis of A549 cells [118]. AgNPs loaded with aqueous pomegranate extract has shown an inhibitory effect in the viability of A549 cells [119].

Furthermore, oral consumption of PE by mice caused a decrease in tumor proliferation in the lungs, compared with control animals [113]. Additionally, A/J mice (cancer model high susceptible to carcinogen-induced tumors) that had received the carcinogenic factor benzo[a]pyrene (B[α]P) and drank water containing 0.2% extract presented a 61.6% reduction in tumors’ volume after 140 days of treatment, while those who had contact with the N-nitrosotris-(2-chloroethyl) urea factor (which induces lung squamous cell carcinoma), showed a reduction in proliferation with treatment after 240 days [114]. Finally, the action of PJ in combination with cisplatin has been studied. It was found that this combination caused a significant increase in the apoptosis of A549 cells, with ideal concentrations of 300 μg/mL of juice and 4 μg/mL of cisplatin [120,121].

### 3.5. Colon Cancer

The first work, based on the study of the effect of pomegranate and tannin juice on colon cancer cells, HT-29, HCT-116, SW-480, and SW-620, showed that punicalagin, ellagic acid and tannins suppressed their proliferation, in a dose dependent manner. In fact, when 100 mg/mL of PJ, ellagic acid, tannins and punicalagin were added, apoptosis was induced in the HT-29 and HCT-116 cell lines [25,122]. Pomegranate fruit exhibited an anti-cancer effect against colon cancer by reducing the number of aberrant crypt foci (ACF) of the colon of male F-344 rats [123]. In literature, it is often stated that ellagitanins, along with urolithins, suppress cell proliferation because they arrest the cell cycle in stages G0/G1 and G2/M, and then lead to apoptosis [124,125]. In addition, ellagic acid and urolithins can detoxify Caco-2 cancer cell enzymes and inhibit the Wnt pathway, which is associated with the development of colon cancer [126]. Pomegranate juice significantly suppressed the expression of COX-2 protein, induced by TNF-α, the action of AKT and the phosphorylation of p65, but also NF-κB factor [127]. In fact, in HT-29 cell lines, PJ reduced COX-2 expression much more effectively than punicalagin, due to the synergistic effect of anthocyanins and flavonols [128]. It has also been demonstrated that punicalin, punicalagin, and granatin B isolated from pomegranate peels reduced HT-29 cells survival by 61.9 ± 4.1%, 86.2 ± 2.2% and 65.3 ± 2.4%, respectively, whereas treatment with granatin B and punicalagin induced S-phase cell cycle arrest and stimulated apoptosis pathways [129]. Pomegranate peel extract has, also, been encapsulated in AgNPs and its activity against colorectal cancer was investigated. Devanesan et al. showed that these nanoparticles reduced the viability of the colorectal cancer cell line ATCC^®^ CRL-2577 at a concentration of 12.5μg [130]. 

In rats, the addition of 0.01% and 0.1% of essential oil suppressed the onset and multiplicity of colon adenocarcinomas and reduced their incidence [131,132]. Additionally, clinical trials conducted with patients suffering colorectal cancer have shown that the consumption of PE for several days, on a daily basis, offsets the changes in the expression of some genes (CD44, CTNNB1, CDKN1A, EGFR, and TYMs) [133]. As a result, it can be concluded that frequent consumption of pomegranate could provide a sufficient concentration of ingredients with a potent activity against the development of colon cancer. 

### 3.6. Other Types of Cancer

Pomegranate is used to prevent cancer, not only in the aforementioned cases, but also in other cancer types. Pomegranate seed extract caused maximum inhibition of SKOV3 ovarian cells by 89%, while peel extract at a rate of 78.2% [79]. Moreover, the addition of 0.125 mg/mL of juice to HeLa cells, a cervical cancer cell line, led to a reduction in their proliferation [134]. Punicalagin, which is contained in pomegranate, exhibits antiproliferative activity against HeLa, while PLE and pomegranate fleshy pericarp extract encapsulated in AgNPs have, respectively, inhibited their growth, with an IC50 value of 100 μg/mL and induced their death [63,135]. The incubation of PE with SKOV3 ovarian cells, HEC-1A endometrial cells, and the SiHa and HeLa cervical cancer cell lines appeared to affect only SiHa proliferation, when the concentration of the extract was 320 μg/mL [136]. Furthermore, ellagic acid appears to act against cancer of the esophagus, intestine, pancreas, bladder, mouth, liver, leukemia, melanoma, and glioblastoma [137]. For example, in glioblastoma, punicalagin reduced the viability of U87MG cells, induced apoptosis and triggered the pathway of caspases 3 and 9 [138]. When ellagic acid was added to WM115 and A375 cells at 40 μM, melanoma cancer cells inhibited their proliferation, migration, and invasion [139].

Concerning oral cancer, ellagic acid added to KB and CAL27 oral cancer cell lines prevented the proliferation of the former by 45–88%, punicalagin by 0–42% and tannins by 0–27%. CAL27 proliferation was reduced by 26–69% in the presence of ellagic acid, 10–96% in the presence of punicalagin, and 17–97% in the presence of tannins [140]. Similar studies have shown that the pomegranate extract exhibited a cytotoxic activity at a concentration of 50 μg/mL in CAL27 cells, 75 μg/mL in SCC1483 and HSC-2 cells and 0–50 μg/mL in HSC-3 and Ca9-22 cells, whereas in normal HF-1 cells the pomegranate extract was cytotoxic at a concentration of 125 μg/mL [141,142]. It was also found that the viability of the cells was reduced, and apoptosis was induced [139]. Additionally, Peng et al. proved that polyphenolic pomegranate extract exerts an apoptotic effect via stimulating mechanisms of mitochondrial impairment. In this study, the application of 24 h ATP assay revealed that the IC50 values of the extract against Ca-22, HSC-2, and OC-2 cancer cell lines were 80.53, 100.34 and 108.12 μg/mL, respectively, thus confirming its strong antiproliferative effect [143]. 

There are also studies related to pancreatic cells. When PANC-1 cells were incubated with PE, their proliferation was prevented, and the cell cycle was arrested. However, a small toxicity in normal cells appeared to be induced, and high concentrations of extract were needed to reduce viability. However, a stronger effect than paclitaxel (a cytotoxic chemotherapy drug) has been shown [144]. Finally, ellagic acid, luteolin, and ursolic acid did not appear to have a synergistic effect, since the compounds alone affect proliferation [144]. In addition, when ellagic acid (10–50 mmol/L) was added in PANC-1 and MIA PACA-2 cell lines, it reduced their proliferation and NF-κΒ activity [145].

Experiments on liver cancer have shown that pomegranate peel polyphenols (100, 200, 300 μg/mL) added in HepG2 cell lines, arrested cell cycle in S-phase [146]. In addition, in mice with dietary carcinogen diethylnitrosamine (DENA)-induced hepatocarcinogenesis that mimics human hepatocellular carcinoma, it has been found that as long as they received DENA alone, they had more tumors than those appeared in combination with pomegranate. Mice that consumed pomegranate had improved liver cell appearance, reduced oxidative damage, and reduced tumor multiplicity [147]. Anthocyanin delphinin, a natural phytotoxin, also causes apoptosis and activates caspase-3 and c-Jun [148]. Last but not least, nanoparticles loaded with pomegranate-derived-punicalagin (100 μg/mL) exert anticancer activity against HepG2 cancer cells inducing a 44% reduction in their viability after treatment [149]. 

Examples of pomegranate parts examined on various cancer cell lines and experimental models and their potential biological effects are summarized in Table 1.

It is clear that there is some discrepancy between the results of studies regarding the health benefits following pomegranate consumption. Under this scope European Food Safety Authority stated a few years ago [150] that no clear cause and effect relationship can be established between consumption of pomegranate and health benefits. This may be due to several reasons including but not limited to different parts of fruit examined and an inter-individual variability regarding the metabolism of pomegranate ellagitannins that leads to the production of urolithins. The health-beneficial effects attributed to pomegranate consumption are linked to the ellagitannins. The ellagitannins are metabolized by intestinal bacteria into ellagic acid analogues called urolithins [151]. Urolithins have been described as the new emerging class of anticancer compounds [152]. In turn the urolithins can be bioavailable and have been reported to accumulate in organs such as the colon, intestines, and prostate [151]. Urolithins are characterized as secondary polyphenol metabolites and as already stated are derived from the gut microbial action on ellagitannins [152]. An essential factor to be considered is the impact of the food matrix and type of ellagitannins on bioavailability and metabolism. Different studies have shown that the nature of ingested ellagitannins could determine the rate of urolithin production [153]. It should be stated that interindividual variability has been reported in people who received pomegranate extract [154].

## 4. Other Healthy Properties

Protection against microbes is an issue of growing concern in the scientific community. Antimicrobials are either synthetic or natural. Natural antimicrobials are usually more efficient than the synthetic ones, as they are able to inhibit more than one microorganism at the same time [155]. Pomegranate contains a variety of phenolic compounds which show natural antimicrobial activity [11]. The decomposition of food and the production of mycotoxins by spoilage fungi cause health problems for consumers and significant financial losses. PP contains a great amount of antifungal compounds and might be a good substitute for synthetic antimicrobials. Specifically, polyphenolic compounds are the reason why PP has a high level of antifungal and antimicrobial properties [156,157]. Punicalagin has been identified as a key component in pomegranate antibacterial action. Punicalagin’s antibacterial activity was investigated against *Candida albicans* and *Candida parapsilosis*, with minimum inhibitory concentrations (MICs) of 3.9 mg/mL and 1.9 mg/mL, respectively. Punicalagin and fluconazole were combined in order to determine their synergistic antibacterial activity. The synergistic affection was shown in time-kill curves created by disk-diffusion and checkerboard experiments, based on fractional inhibitory concentration (FIC), which is defined as the sum of MIC related to a drug divided by the MIC of the drug employed alone. Punicalagin had a reported FIC of 0.25 [158]. Antimicrobial activity of pomegranate peel hydro-alcoholic extract against *Bacillus subtilis*, *Staphylococcus aureus* and *Pseudomonas aeruginosa* was found with MIC values ranging from 0.25 to 0.89 mg/mL [159]. *Bacillus megaterium*, *Bacillus subtilis, Staphyloccocus aureus* and *Pseudomonas sp*. were found to be sensitive to juice samples [160]. The fruit variety is another important factor in pomegranate’s antibacterial activity. Rosas-Burgos et al. studied the antibacterial affinity of six cultivars against *E. coli*, *Shigella sonnei*, *Salmonella enterica*, *Bacillus subtilis*, *Enterococcus faecalis*, and *Staphylococcus aureus* [161]. Ellagic acid was found effective against *Salmonella enterica*, *Shigella sonnei*, *Escherichia coli*, *Enterococcus faecalis*, *Bacillus subtilis*, and *Staphylococcus aureus* [161]. Gullon et al. investigated the antibacterial properties of the pomegranate variety “Mollar de Elche” against *Listeria monocytogenes*, *Listeria innocua*, *Staphylococcus aureus*, *Pseudomonas aeruginosa*, *Escherichia coli*, and *Salmonella enterica* [162]. Pomegranate peel flour minimized *Salmonella enterica*, *E. coli*, *Staphylococcus aureus*, and *Listeria monocytogenes* [162]. Against *Salmonella mutans, Salmonella mitis*, and *Lactobacillus acidophilus*, pomegranate peel extracts had stronger antibacterial activity than blossom, leaf, and stem extracts [163]. Similarly, *Staphylococcus aureus*, *Staphylococcus epidermidis*, *Lactobacillus acidophilus*, *Actinomyces viscosus*, *Streptococcus mutans*, *Streptococcus sanguinis*, and *Streptococcus salivarius* [164], *Listeria monocytogenes*, *Yersinia enterocolitica*, *Escherichia coli*, and *Staphylococcus aureus* [165]. Antimicrobial activity has been shown in phenolic compounds isolated from pomegranates. Pagliarulo et al. studied the bioactive polyphenols (mainly tannins, catechins, gallic and ellagic acid, and anthocyanins) in pomegranate from the Campania Region in Southern Italy. The antibacterial activity was tested using agar diffusion techniques, and the results revealed that it was effective against *Staphylococcus aureus* and *Escherichia coli* [166]. Water, methanol, and ethanol extracts of pomegranate peel powder were found to be effective against *Salmonella enterica* and *Salmonella kentucky*, respectively [167]. Punicalagin isomers and bishexahydroxydiphenoyl-glucoside isomer were reported to have the strongest antibacterial action among the discovered phenolic compounds, the majority of which had antimicrobial activity [168].

## 5. Pomegranate, Animal Nutrition and Food Applications

### 5.1. Animal Nutrition

The exploitation of pomegranate by-products as feed additives appears to be a promising strategy to improve waste valorization and supply animals with bioactive compounds capable to improve animals’ oxidative status and products’ oxidative stability. Several studies revealed promising results following pomegranate addition to animal diets. Most notably, partial replacement of cereal grains of kid’s diet by pomegranate seed pulp at 150 g/kg led to a greater antioxidant capacity and lower MDA concentration than that of control diet fed kids [169]. In support of valorization strategies, the aforementioned study indicated that pomegranate seed pulp was found to be a cost-effective antioxidant rich feedstuff in ruminant production since inclusion in kid’s diet decreased cost of meat production [169]. In lambs, dietary pomegranate by-product silage supplementation improved meat quality characteristics and antioxidant potential, as indicated by the increase of essential fatty acids, linoleic, α-linolenic, and in trans-10, cis-12 CLA fatty acids present in intramuscular fat accompanied by an increase in total phenolic content and antioxidant activity [170]. In dairy cows, inclusion of pomegranate pulp silage up to 150 g/kg dry matter in the total mixed ration did not affect milk yield and chemical composition but improved the milk FA profile and blood plasma antioxidant status [171]. Most notably, the same authors reported that as dietary pomegranate pulp silage inclusion levels increased medium chain FA, saturated FA (SFA), the SFA/UFA ratio and atherogenicity index significantly decreased, whereas long chain FA, monounsaturated FA (MUFA) and polyunsaturated FA (PUFA) significantly increased. In broilers, pomegranate by-product dietary supplementation (from 0.5 up to 2%) reduced lipid oxidation values and SFA and increased MUFA, PUFA and n-3 fatty acids in meat thus pomegranate by-products improved the meat composition and fatty acid profile of broiler meat [172]. A study with pomegranate seed oil addition to laying hen diets revealed that pomegranate seed oil, a rich source of punicic acid, positively affected the yolk color of eggs, and in addition, increased not only the punicic acid concentration in egg-yolk lipids but also conjugated linoleic acid, a fatty acid known to exert several health benefits [173].

### 5.2. Food Applications

Nowadays, there is an increasing interest in the consumption of different food types originating from all around the world. For this reason, food industry has made a great effort to prolong the shelf-life of food. To that end, food additives of both natural and synthetic origin are widely used in the food industry as preservatives [174]. However, there is a great concern regarding the use of synthetic additives in food preservation since these compounds are linked with toxicity posing a risk for human health [10]. Therefore, lately there has been a growing research interest for antimicrobial compounds of plant origin, which decompose easily and do not cause toxicity to the environment. These compounds have the ability to inhibit food-borne pathogens and extend the shelf-life of perishable foods. 

In addition, in many fruits and vegetables, herbal plants included as well, there are compounds which can contribute to the reduction in many food-related diseases. Especially, fruit peels contain compounds with antimicrobial- and antioxidant activity. These compounds are secondary metabolites: phenolic compounds (e.g., vitamins, flavonoids), steroids and alkaloids. Use of these natural originated compounds as food additives extend food life and reduce the possibility of foodborne illness [175].

Pomegranate is a fruit-bearing-plant that is well known for its medicinal properties. One of the byproducts that derive from the production of pomegranate juice (PJ) is pomegranate peel (PP) [174]. Pomegranate Peel is considered a good source of phenolic acids, tannins (ellagitannins, such as punicalin and punicalagin) and flavonoids [160]. These compounds have attracted the interest of many scientists and could constitute a promising option in the development of new antimicrobials [12,176]. PP ellagitannins containing phenolic hydroxyl groups transfer hydroxyl residues to free radicals. As a result, these harmful species become quenched [177]. Generally, the relative potency of PP extract flavonoids to act as antioxidants is attributed to the number and configuration of hydroxyl groups. In 2014, Karaaslan et al. proved that in contrast with anthocyanins, flavonoids and phenolic acids are mostly responsible for pomegranate’s bioactivity [178]. In addition, in 2014, Cam et al. showed that PP phenolics can be microencapsulated and constitute potential ingredients for functional food development [179]. Microencapsulation, a good option to stabilize phenolics, is defined as a process whereby tiny particles are surrounded by a coating or embedded in a homogeneous or heterogeneous matrix in order to produce small capsules with useful properties [180]. Another study demonstrated antibacterial- and antifungal activity of PP [181]. In short, due to its components, PP could be used in the food industry as an antioxidant and antimicrobial agent.

Although PP is richer in phenolic content than pomegranate juice [182], PJ also contains a variety of phenolic compounds (anthocyanins, tannins, and phenolic acids) and has antioxidant properties [183]. Pomegranate seed is another by-product that derives from the PJ production, which contains crude protein, crude lipids, dietary fiber, minerals, and phenolic compounds and shows antioxidant activity [184]. For these reasons, PJ and pomegranate seed are also possible ingredients that could be used in the food industry to improve the food quality.

#### 5.2.1. Beef Meat

Turgut et al. (2017) studied the antioxidant effect of PP to delay lipid and protein oxidation in beef meatballs through chilled storage at −18 ± 1 °C. Freeze dried and concentrated aqueous extract of pomegranate peel was added into freshly prepared meatball mix at 0.5% and 1.0% concentrations, and then compared to 0.01% butylated hydroxyl toluene (BHT) and negative controls. Six months of storage later, peroxide, loss of total protein solubility, malondialdehyde, and carbonyl formation and sulfhydryl groups were remarkably lower in PP at 1.0% than control samples. This high concentration of PP (1%) also led to maintenance of its color intensity and hue value. The results from sensory analyses proved that PP addition to meatballs was effective on preventing rancid smell [185]. In another study, Turgut et al. (2016) [186] added PP extract at concentrations of 0.5% and 1% in Turkish meatballs and studied its impact on delaying lipid and protein oxidation after 8 days in chilled storage at 4 ± 1 °C. The addition of concentrated lyophilized pomegranate extract at concentrations of 0.5% and 1% in meatballs resulted in reduction in lipid and protein oxidation and in improvement of sensory scores. After the storage period, peroxide formation, thiobarbituric acid reactive substances (TBARS) value, loss of sulfhydryl groups and formation of protein carbonyls were lower in treated samples than in negative (without any antioxidant) and positive (BHT at 0.01%) controls (*p* < 0.01). Color and rancid smell showed that PP incorporation in meatballs extended the refrigerated storage up to 8 days [186].

#### 5.2.2. Poultry Meat

Under this scope, Kanatt et al. (2010) studied the effect of aqueous PP extract on the shelf-life of commercially prepared chicken chilly and chicken lollipop. The concentrations were 0.1% and 0.5% [187]. In fact, the addition of pomegranate extract to Indian chicken meat products increased its shelf-life by 2–3 weeks while being in chilled storage. *Staphylococcus aureus* was detected in treated chicken lollipop only after 12 days of storage while in chicken chilly untreated control samples it was detected within 7 days of storage. No fecal coliforms were detected. Oxidation, which was defined by TBARS, was in lower levels in chicken chilly and lollipop containing aqueous PP through the storage period compared to untreated control samples. After 20 days of chilled storage, chicken chilly and lollipop of 0.5% PE concentration was sensory acceptable. Naveena et al. (2008) [188] reported a significant reduction in TBARS of 10 mg tannic acid equivalent phenolics/100 g in fresh chicken in comparison to positive (BHT) and negative control samples (meat treated without antioxidants). 

Naveena et al. (2008) [189] reported a reduction (*p* < 0.05) in TBARS of cooked chicken patties treated with 10 mg equivalent RP (rind powder extract) phenolics per 100 g meat, in comparison to positive (100 mg BHT per 100 g meat) and negative control samples (treated with no antioxidants). In this case, 15 days later, while being at 4 °C of storage, TBARS values (0.203 mg malonaldehyde/kg) of treated samples were 15.6 (0.203/1.272) and 22.56% (0.203/0.896) times lower than those of negative control and BHT samples, respectively [159]. In the same study, it was reported that regarding instrumental color values, in comparison to control samples, the pomegranate rind powder extract treatment significantly decreased the lightness values on the surface of cooked patties and increased redness values. Nonetheless, no difference was noticed in the internal redness values between samples. In contrast to instrumental color values, sensory evaluation levels did not reveal a great difference in appearance between treated and control samples. No difference was observed in smell, sweet flavor, chicken flavor, and overall palatability levels [189]. The pomegranate rind powder extract at 5, 10, 15 and 20 mg equivalent rind powder phenolics/100 g of cooked chicken patties, in comparison to vitamin C, preserved cooked chicken patties during refrigerated storage [188]. Moreover, the incorporation of pomegranate rind powder extract into chicken patties significantly decreased the lightness values compared to negative and positive control samples. Lipid oxidation was substantially inhibited in cooked chicken patties to a much greater extent than vitamin C treatment [188]. Similarly, 2 g/kg of pomegranate peel powder meal added to broiler diets significantly improved the water-binding capacity of chicken breast meat due to the reduced cooking loss of meat [190]. Suresh Devatkal et al. (2014) applied 1% pomegranate peel extract to reduce the lipid oxidation in pressure-treated chicken nuggets. The results revealed that this extract could be a decent choice of natural antioxidants for pressure-treated meat products [191].

#### 5.2.3. Goat Meat

Devatkal and Naveena studied the effects of salt, kinnow and pomegranate by-product powders on color and oxidative stability of raw ground goat meat stored at 4 ± 1 °C [192]. In this study, 5 treatments were evaluated: control (only meat), MS (meat + 2% salt), KRP (meat + 2% salt + 2% kinnow rind powder), PRP (meat + 2% salt + 2% pomegranate rind powder), and PSP (meat + 2% salt + 2% pomegranate seed powder). During 6 days of refrigerated storage, there was an increase in lightness for the control sample and a decrease in lightness for the MS treatment. However, lightness in the other treatments remained unchanged. In the meantime, redness levels of all treatments declined, and yellowness showed inconsistent changes. The lipid oxidation, during 6 days of refrigerated storage, was evaluated by calculating the overall percent increase in TBARS of different treatments. The highest percentage increase in TBARS was shown in the MS treatment followed by control, PRP, KRP, and PSP. The results of this study indicate the oxidative effect of salt and the antioxidant effect of KRP, PRP, and PSP in goat meat. The authors suggest the use of kinnow and pomegranate fruit powders in raw ground goat meat as natural antioxidants [192]. Devatkal and Naveena (2010) used pomegranate rind powder (PRP), kinnow rind powder (KRP), and pomegranate seed powder (PSP) extracts in goat meat patties. Lightness value lowered remarkably (*p* < 0.05) in PRP during 12 days in refrigerated storage as compared to control, followed by PSP and KRP patties. Sensory evaluation did not show significant differences (*p* > 0.05) among different treatments. However, a significant (*p* < 0.05) reduction in TBARS values (lipid oxidation) during storage of goat meat patties was noticed in PRP, PSP, and KRP in comparison to control patties [192]. Another study investigating the effects of dietary pomegranate seed pulp on oxidative stability of kid meat revealed that replacement of barley and corn grains with PSP in the diet improved the color and lipid stability of kid meat [193]. Most notably, meat from kids fed the PSP had higher color stability than that of kids fed the control diet. In addition, the meat color deterioration was less in the *longissimus lumborum* muscle during the storage time for kids fed 150 g/kg pomegranate seed pulp in the diet compared to control [193]. It seems that improvement of selected stored meat product quality indices including but not limited to instrumental color retaining, limitation of microflora growth, retardation of lipid, and protein oxidation can be attributed to pomegranate peel phenolics [194]. 

Similarly, another study pointed out that encapsulated or even crude pomegranate peel extracts may increase stability of foods upon processing and storage by inhibiting oxidation and growth of pathogenic microorganisms [10].

#### 5.2.4. Dairy Products

Pomegranate juice, in the form of powder, can be used to make yogurt, mostly as a sugar replacement. Pomegranate juice powder (5%) was added to produce a product with higher total phenolic content, antioxidant activity, and in vitro bio-accessibility. Furthermore, due to phenolic–protein interactions, it has been demonstrated to positively alter the product’s sensory qualities, resulting in a more solid-like behavior [195]. In a similar trial with kefir-like products, adding fresh juice (5%) boosted viscosity and acidity, but necessitated the addition of honey to improve sweetness [196]. Another option to include pomegranate’s health benefits into dairy products is to use pomegranate peel extract. This extract is mostly utilized to boost a product’s antioxidant activity as well as its storage shelf-life [197,198]. It has also been employed in the manufacture of cheese with better lipid oxidative stability and storage quality [199]. However, due to its high astringency and bitterness, the use of this extract may cause undesirable changes in the sensory features of the products [198]. In general, pomegranate peel extract can be utilized as a potential natural preservative in fermented dairy products, albeit it should be used in small amounts to prevent adverse influence on sensory properties. As in the case of freeze-dried yogurt [200], the inclusion of honey may minimize these effects and improve the appeal of these products. Pomegranate seed powder, which is high in conjugated linolenic acids, is used in dairy products such as yogurt. Yogurt enhanced with 0.5% (*w*/*v*) pomegranate seed powder had comparable nutritional and pH values, better antioxidant activities, acceptable fatty acid and conjugated linolenic acid levels, and reduced atherogenicity indexes when compared to the control sample [201]. When pomegranate peel phenolics were added to ice cream at quantities of 0.1 and 0.4% *w*/*w*, substantial changes in pH, total acidity, and color were observed. The most important consequence of the phenolics incorporation was a significant increase in antioxidant and antidiabetic activity, as well as phenolic content in ice creams [202]. Microencapsulated pomegranate peel phenolics were explored by Cam et al. [179] to improve the functional qualities of ordinary ice cream. The antioxidant and a-glucosidase inhibitory activity of the enriched ice creams were significantly improved when pomegranate peel phenolics were added at 0.5 and 1.0% (*w*/*w*) compared to the control sample. Chan et al. [197] investigated the antioxidant activity of *Lactobacillus acidophilus*-fermented milk fortified with freeze-dried PPE at concentrations ranging from 0.5 to 4.0% (*w*/*v*). They found that PPE at 2% had no effect on *L. acidophilus* growth, implying that it had no influence on cell viability during milk fermentation, although total phenolic content and radical scavenging capacity of the hydrophilic portion of the milk rose after fermentation. Fermented milk beverages supplemented with dry aqueous PPE (150 and 300 mg/L) and colonized with *Lactobacillus plantarum* and *Bifidobacterium longum* were examined. In vivo, the fortified products were effective in lowering LDL cholesterol and triacylglycerol while boosting HDL cholesterol in albino male rats’ serum, while TBARS dropped and antioxidant enzyme activity in the liver increased. These findings suggest that fermented dairy products with additional phenolics from pomegranate peels could be a good food system for providing probiotic effects as well as the health advantages of added antioxidants [203].

#### 5.2.5. Fish Products

Basiri et al. [204] investigated the impact of vacuum packaging and a methanolic PPE on the shelf-life and quality of Pacific white shrimp during refrigerated storage (4 °C) for 10 days. The researchers examined changes in pH, TBARS, trimethylamine, aerobic plate counts, lactic acid bacteria, *Enterobacteriaceae* count, and melanosis. Additionally, professional panelists conducted sensory evaluations of the food after cooking, both with and without treatment. PPEs (1 or 2% *w*/*w*) combined with vacuum packaging could act as antibacterial agents, improve shrimp quality, and extend shelf-life. Color, odor, texture, and overall similarity scores for shrimp treated with PPE were high after 10 days of storage, which was attributable to lesser melanosis in these samples. The authors found no evidence that the PPE’s sensory qualities had a detrimental impact on the product’s overall acceptance. Aqueous methanol extracts from pomegranate peel and Chinese gallnut were utilized by Wu et al. [205] to suppress the foodborne pathogenic microorganisms *Vibrio parahaemolyticus* and *Listeria monocytogenes* in cooked shrimp and raw tuna. Pomegranate peel and Chinese gallnut extracts significantly suppressed the growth of *V. parahaemolyticus* in shrimp and tuna, but only the latter inhibited the growth of *L. monocytogenes*, according to the researchers. However, neither of the extracts was shown to entirely stop the bacteria from growing. In terms of sensory properties, the scientists reported that both studied extracts whitened the tuna meat, and that more research in this area is needed. PPE in combination with Modified Atmosphere Packaging (MAP) (40% CO_2_, 60% N_2_ and 30% CO_2_, 10% O_2_, 60% N_2_) was used to extend the shelf-life of *Litopenaeus vannamei* during 24 days of chilled storage, according to Udayasoorian et al. [206]. The authors concluded that using PPEs in combination with MAP increased the shelf-life of *L. vannamei* from 4 to 24 days under refrigeration without synthetic preservatives, as measured by trimethylamine nitrogen (TMA), total volatile base nitrogen (TVB), TBARS, total plate count (TPC), psychrotrophic counts, and hypoxanthine analysis. Marn et al. [207] encapsulated an ethanolic PPE in phosphatidylcholine liposomes and included the freeze-dried liposomes at a concentration of 10.5% in squid surimi gels. The inclusion of freeze-dried liposomes resulted in a modest loss in gel strength, but it helped to keep the gels stable during long-term storage. Furthermore, encapsulated PPEs were found to have a reduced impact on the color of the food product, as determined by colorimetric analyses, than non-encapsulated PPEs employed as a control.

#### 5.2.6. Shelf Life

Ιn Europe, antioxidants are considered to be “substances which prolong the shelf-life of foods by protecting them against deterioration caused by oxidation, such as fat rancidity and color changes” [208]. On the other side of the coin, according to the Institute of Medicine (US), “a dietary antioxidant is a substance in foods that significantly decreases the adverse effects of reactive species, such as reactive oxygen and nitrogen species, on normal physiological function in humans” [209]. In the food industry, antioxidants (E300–E399) are mostly used in order to extend food shelf-life as they delay oxidation of lipids and vitamins in food, by preventing their autoxidation and, therefore, the development of rancidity or other unwanted flavors [210]. The use of PP, or even its extracts, which have antioxidant and anti-food borne pathogen bacteria properties, can be a good alternative to replace the use of the synthetic antioxidants (e.g., BHA, BHT, TBHQ, and PG) in food. Furthermore, the use of these natural ingredients would possibly increase the quality and the shelf-life of meat [194]. In modern society, people may prefer foodstuff of increased shelf-life. The food industries have been trying to find the most appropriate way to make it possible. Food additives, such as preservatives, either synthetic or of natural origin may preserve freshness and increase shelf-life [174]. However, in the recent years, the meat industry actively pursues antioxidants from natural sources mostly because of adverse toxicological reports on several synthetic compounds. Pomegranate and its by-products exhibit a potential for perspective application in meat products as they present strong antioxidant and antimicrobial activities. Under this context, a review on the incorporation of pomegranate peel in meat products revealed that this is a suitable strategy to enhance meat product quality [211]. The same study also pointed out that this approach could open a new field in the meat sector since it can create functional meat products tailored to the consumers demands [211].

#### 5.2.7. Edible Oils

PPEs, which are high in polyphenols, could potentially be used to replace synthetic antioxidants in a variety of edible oils and fats, including sunflower oil, corn oil, coconut oil, palm oil, sesame oil, and peanut oil. Iqbal et al. [212] investigated the effect of different concentrations (250, 500, and 1000 ppm) of a methanolic PPE on the stabilization of sunflower oil under accelerated conditions (i.e., storage at 65 °C for 4 days in the presence of oxygen) versus BHT (200 ppm). The addition of 1000 ppm PPE stabilized sunflower oil in terms of antioxidant activity index, peroxide value, conjugated dienes and trienes, as well as TBARS. In addition, Mekawi et al. [213] compared the effects of a lyophilized aqueous ethanolic PPE (1000 ppm) on the oxidative stability of sunflower oil and the decrease of acrylamide production during deep-frying of potato chips to BHT (200 ppm) and tocopherols (1000 ppm). PPE could be employed as a natural antioxidant to improve the oxidative stability of sunflower oil during deep-frying or thermal processing. Bashir et al. investigated the stability of sunflower oil during the frying of chicken nuggets [214]. Ethanolic and methanolic PPEs were added to sunflower oil (500, 750, and 1000 ppm, respectively), which was subsequently used to cook chicken nuggets. As a control, 200 ppm BHT was employed. The scientists found that PPEs ha antioxidant properties that may be used to extend the shelf-life of fatty foods. Furthermore, sensory examination of cooked chicken nuggets in terms of color, taste, texture, softness, and crispiness revealed that the general acceptability of chicken nuggets produced with sunflower oil, treated with either PPEs or synthetic antioxidant, was identical. The stability of sunflower oil following addition of ethanolic and methanolic PPEs (500, 750, 1000 ppm) and storage for 30 days was also tested by the same research group [215]. In comparison to the control sample, which was supplemented with 200 ppm BHT, the authors discovered that adding the extracts at the maximum level (1000 ppm) resulted in a decrease in free fatty acids, iodine value, saponification value, peroxide value, and TBARS. The effect of a PPE added at 2% (*w*/*w*) on the oxidative stability of several edible oils, such as sunflower oil, coconut oil, palm oil, and sesame oil, during deep frying of potato strips at 170 °C was also examined [216]. In comparison to the corresponding untreated controls, there was a reduction in oxidation of the tested oils after three repeated frying cycles in terms of peroxide value and TBARS. The scientists concluded that sesame oil, which has the highest level of unsaturated fatty acids, was the least resistant to oxidation.

#### 5.2.8. Cookies, Cereals and Nut Products

Kaderides et al. [217] showed that pomegranate peel extract (PPE), either crude or encapsulated, improved the shelf-life of hazelnut paste by inhibiting lipid oxidation. In 2019, Kaderides et al. [218] enhanced cookies with crude and encapsulated PPE. Although the baking procedure reduced the total phenolic content and antioxidant activity of fortified cookies by 65 and 76% for encapsulated and crude extract, respectively, the total phenolic content and antioxidant activity of fortified cookies remained higher than control samples throughout the 21-day storage period at 25 °C. In general, cookies with encapsulated extract had higher total phenolic content and antioxidant activity than cookies with crude extract, indicating that encapsulation had a protective impact. Furthermore, the addition of extracts to cookies had no negative impact on their sensory properties. Pomegranate seed powder was used to boost the total phenolic content and antioxidant activity of gluten-free bread [219]. Pomegranate seed powder boosted the specific volume and springiness of gluten-free loaves but lowered their hardness and chewiness dramatically as powder additions increased. There were also reports of a decrease in the brightness and yellowness of crumb and crust color, as well as an increase in redness. In general, using 7.5% pomegranate seed powder produced the finest gluten-free bread with the greatest physical attributes and excellent antioxidant capacities. Pomegranate seed powder has also been used to make gluten-free cake [220] and gluten-free sheeted pasta [221], following this trend. Gluten-free cake with 25% pomegranate seed powder and 0.97% transglutaminase had better overall antioxidant activity, ash, fiber, protein, and moisture content, as well as reduced peroxide value, volume index, and porosity [220]. Although there was an increase in antioxidant activity in the case of sheeted pasta, the addition of pomegranate seed powder influenced cooking and textural characteristics. In average, the lowest concentration of pomegranate seed powder had the least effect, therefore gluten-free pasta with up to 7.5% pomegranate seed powder had high acceptance [221].

#### 5.2.9. Active Food Packaging

The addition of PP extracts in meat products might be indirect by putting these extracts in active food packaging. For this reason, Licciardello et al. [222] evaluated the prospect of edible coatings based on PP extract and chitosan to maintain quality of shrimps during refrigerated storage. In vivo trials were performed on headed and peeled shrimps covered with chitosan and PP extract to assess the effect throughout storage of different coatings on *Pseudomonas spp.* and total psychrotrophic bacteria counts. Total Volatile Basic Nitrogen (TVBN) and visual color were evaluated as quality indexes. The results revealed the effective synergy of PP extract with chitosan at decreasing microbial spoilage during storage, reducing the *Pseudomonas* spp. counts by almost 2 Log units and at maintaining the psychrotrophic microbial load under 7 LogCFU/g for 6 days. Furthermore, after 6 days, TVBN levels in shrimps covered with chitosan and PP extract were as low as in the control samples after 2 days. Hu et al. [223] studied the effects of mono and double-layer active films, which contained PP extract, on pork stored at refrigeration. Firstly, two different monolayer films were prepared, the one containing only polyethylene (PE) resin and the other containing a combination of polyethylene resin with 1.5% of PPE. Using the PE and the PPE-PE layers as internal layers two double-layer active films, PPE-PE/polyethylene terephthalate (PET) and PPE-PE/polypropylene (PP), were obtained. Evaluation of the levels of TVBN, TVC (total viable counts), pH and TBARS in the pork was conducted. TVC values in PPE-PE, PPE-PE/PP, PPE-PE/PET, and PE/PET treatments were significantly lower than those in the control PE group during the storage of days 2–6. Furthermore, the application of PPE-PE, PPE-PE/PP, PPE-PE/PET, and PE/PET films retarded the spoilages in pork, as indicated by pH, TVBN, TBARS and sensory scores. Especially PPE-PE/PP and PPE-PE/PET active films showed the best activity against both microbial and chemical spoilage and could extend pork shelf-life by 2–3 days in refrigeration.

## 6. Conclusions

Cancer is among the most prevalent diseases encountered in developed and developing countries presenting high death rates worldwide. In this review, the potential anticancer properties of various phytochemicals, derived from different anatomical parts of pomegranate, including juice, peels, and seeds, against different types of cancer, were examined. A plethora of clinical trials conducted in men with prostate cancer have demonstrated that the consumption of PJ or PE could be promising against prostate cancer, as it showed a significant effect in decelerating PSA doubling time. However, there is still need in conducting well-designed, properly powered, randomized, and controlled clinical trials in other cancer types, such as breast, skin, lung, and colon cancer, where the obtained results from in vitro and in vivo studies indicate the effectiveness of pomegranate components against cancer cell growth, proliferation, and metastasis. Furthermore, the encapsulation of the bioactive components in AuNPs, AgNPs and PtNPs for the optimization of their bioavailability and targeted transport, leads to an inhibition of cell proliferation and reduces the survival rates in breast, lung, and colon cancer cell lines. Additionally, both clinical trials and in vivo studies in mice and rats have indicated that pomegranate consumption has no significant adverse effects in health, further highlighting the important anticancer activity of pomegranate. The detailed biochemical and molecular pathways which are followed by pomegranate’s constituents to exert their beneficial effects on various types of cancer remain unknown.

The future regarding pomegranate use in human and animal nutrition is promising but new coordinated strategies between healthcare professional, policy makers, academia and industry are needed to address any data discrepancy and design limitations of previous studies. This is supported by the EFSA conclusions, a few years ago, that there is no sufficiently well-established cause and effect relationship between the consumption of pomegranate-derived products and health beyond basic nutrition [150]. Future studies aiming, by pomegranate supplementation, to ameliorate disease detrimental symptoms will provide valuable findings and potential therapeutic schemes. In the meantime, animal studies elucidating the potential effect of pomegranate supplementation in antioxidant function and overall therapeutic efficiency are of great value. Moreover, the reuse of pomegranate by-products may contribute to a circular economy model and sustainability.

## Figures and Tables

**Table 1:** Examples of pomegranate parts examined on various cancer cell lines and experimental models and their potential biological effects.

Formulation	Biological Effect	Cancer Cell Line/Experimental Model	References
Peel extract	Apoptosis induction, inhibition of tumor cell growth and proliferation, cell cycle arrest, survival reduction, cytotoxicity, inhibition of UV-B action and angiogenesis, survival suppression	MCF-7, WA4, BT474, MDA-MB-231, SiHa, CAL27, SCC1483, HSC-2, A549, PC-3, B[α]P-induced lung cancer in mice, fibroblasts, against Ca-22, HSC-2, OC-2	[36,37,38,39,40,41,42,46,47,48,50,71,79,114,134,136,141,142,143]
Homogenized pomegranate peel	Inhibition of tumor cell proliferation	MCF-7, MDA-MB-453, LNCaP	[49]
Pomegranate peel polyphenols	Cell cycle arrest	HepG2	[146]
Peel	Apoptosis induction, suppression of tumor growth	TRAMP-C1 mice	[86]
Consumption of peel extract	Decreased number of tumors, decrease in androgen signaling, decrease in the expression of biomarkers related to oxidative stress, offset of the changes in the expression of some genes	SKH-1 hairless mice with skin cancer, patients with prostate cancer, patients with colorectal cancer	[89,105,106,107,113,133]
Oil and phenols from pericarp	Suppression of tumor cell proliferation	LNCaP, PC-3, DU145	[75]
Oil from fruit	Reduction in rate of tumor formation and multiplicity	DMBA-induced skin carcinogenesis in CD1 mice	[108]
Essential oil	Multiplicity suppression	Colon adenocarcinomas	[131,132]
Juice	Growth inhibition, suppression of tumor cell proliferation, inhibition of estrogens, apoptosis induction, chemotaxis, and metastasis reduction through SDF1a pathway, increase PSA’s doubling time,	MCF-7, MDA-MB-231, PC-3, DU145, LNCa-P, HeLa, patients with prostate cancer	[52,68,90,91,92,93,96,134]
Juice + ellagic acid + tannins + punicalagin	Apoptosis induction	HT-29, HCT-116, SW-480, SW-620	[25,122]
Juice + cisplatin	Apoptosis induction	A549	[120]
Ellagic acid + punicalagins from juice	Growth inhibition	LNCaP, LNCap-AR, DU145	[74]
Ellagitannins + ellagic acid from extract	Suppression of tumor cell proliferation	HUVEC, LNCaP	[77]
Punicalagins + ellagic acid from peel extract	Suppression of tumor growth, decrease of NF-κB activity	SCID mice infected with LAPC4	[84]
Ellagic acid + urolithin A from extract	Suppression of tumor growth	Mice infected with LAPC4, LNCaP, DU145, 22RV1	[85]
Polyphenols of the whole fruit, pericarp, and seeds	Anti-proliferative properties, prevention from invasion through Matrigel, increase of G2/M ratio	LNCaP, PC-3, DU145	[64]
Hydrophilic fragments of pomegranate oil	Survival suppression	MDA-MB-231, MCF-7	[57]
Roots, bark, fruit	Inhibition of tumor cell proliferation	MCF-7	[58]
Peel and folic acid in AuNPs	Cytotoxic activity	MCF-7	[66]
Fleshy pericarp extract encapsulated in AgNPs	Growth inhibition	MCF-7, HeLa	[63,135]
Extract encapsulated in PtNPs	Inhibition of tumor cell proliferation	MCF-7	[60]
Extract encapsulated in AgNPs	Inhibition of tumor cell proliferation, cytotoxic activity, reduction in cell viability	MCF-7, MDA-MB-231, A549, ATCC^®^ CRL-2577	[61,62,63,64,119,130]
Extract encapsulated in chitosan-coated magnetic nanoparticles	Cytotoxic activity	4T1, MBA-MB-231, NIH/3T3	[65]
Extract encapsulated in solid lipid nanoparticles	Tumor size reduction	Mice with EAC	[110]
Ellagic acid + punicic acid + luteolin	Growth inhibition, apoptosis induction, chemotaxis, and metastasis reduction through SDF1a pathway	MDA-MB-231, MCF-7, SCID mice, PC-3, DU145, LNCaP	[52,87]
Ellagic acid + urolithins	Detoxification, inhibition through Wnt pathway, growth reduction	Caco-2^+^, LAPC4, LNCaP, DU145, 22RV1	[126]
Punicalagin from pomegranate fruit extract	Survival suppression, reduction in expression of D1, D2, E, cdk2, cdk4, cdk6,Cytotoxic activity	A549, MCF-7	[44,117]
Punicalagin encapsulated in nanoparticles	Cell viability reduction	HepG2	[149]
Punicalagin, punicalin and granatin B	S-phase cell cycle arrest, cell survival reduction	HT-29	[129]
Pure ellagic acid	Inhibition of tumor cell proliferation, changes in morphology, decrease of NF-κB activity, suppression of migration, invasion, and proliferation	LNCaP, PC-3, KB, CAL27, WM115, A375, PC-3, PANC-1, MIA PACA-2	[70,72,73,140,145]
Pure ellagitannins	Suppression of Bcl2, apoptosis induction	A549	[118]
Pure punicalagin	Apoptosis induction, reduction in proliferation	LNCaP, PC-3, HeLa, U87MG, CAL27, KB	[63,82,135,138,140]
Pure urolithin A	Cytotoxic activity	DU145, PC-3	[83]
Pure delphinidin	Inhibition of tumor cell proliferation	HCC180, MDA231, MDA468, SKBR3, MDA453, BT474, MCF7	[56]
